# Boosting Genetic Gain in Allogamous Crops *via* Speed Breeding and Genomic Selection

**DOI:** 10.3389/fpls.2019.01364

**Published:** 2019-11-15

**Authors:** Abdulqader Jighly, Zibei Lin, Luke W. Pembleton, Noel O. I. Cogan, German C. Spangenberg, Ben J. Hayes, Hans D. Daetwyler

**Affiliations:** ^1^Agriculture Victoria, AgriBio, Centre for AgriBiosciences, Bundoora,VIC, Australia; ^2^School of Applied Systems Biology, La Trobe University, Bundoora,VIC, Australia; ^3^Queensland Alliance for Agriculture and Food Innovation, Centre for Animal Science, University of Queensland, QLD, Australia

**Keywords:** allogamous breeding, inbreeding, genomic selection, simulation, speed breeding

## Abstract

Breeding schemes that utilize modern breeding methods like genomic selection (GS) and speed breeding (SB) have the potential to accelerate genetic gain for different crops. We investigated through stochastic computer simulation the advantages and disadvantages of adopting both GS and SB (SpeedGS) into commercial breeding programs for allogamous crops. In addition, we studied the effect of omitting one or two selection stages from the conventional phenotypic scheme on GS accuracy, genetic gain, and inbreeding. As an example, we simulated GS and SB for five traits (heading date, forage yield, seed yield, persistency, and quality) with different genetic architectures and heritabilities (0.7, 0.3, 0.4, 0.1, and 0.3; respectively) for a tall fescue breeding program. We developed a new method to simulate correlated traits with complex architectures of which effects can be sampled from multiple distributions, e.g. to simulate the presence of both minor and major genes. The phenotypic selection scheme required 11 years, while the proposed SpeedGS schemes required four to nine years per cycle. Generally, SpeedGS schemes resulted in higher genetic gain per year for all traits especially for traits with low heritability such as persistency. Our results showed that running more SB rounds resulted in higher genetic gain per cycle when compared to phenotypic or GS only schemes and this increase was more pronounced per year when cycle time was shortened by omitting cycle stages. While GS accuracy declined with additional SB rounds, the decline was less in round three than in round two, and it stabilized after the fourth SB round. However, more SB rounds resulted in higher inbreeding rate, which could limit long-term genetic gain. The inbreeding rate was reduced by approximately 30% when generating the initial population for each cycle through random crosses instead of generating half-sib families. Our study demonstrated a large potential for additional genetic gain from combining GS and SB. Nevertheless, methods to mitigate inbreeding should be considered for optimal utilization of these highly accelerated breeding programs.

## Introduction

Allogamous species are obligate outbreeding, or cross-pollinated, due to the presence of self-incompatibility mechanisms that cause dissonant pollen-stigma interactions (McClure et al., 1989). Allogamous species include a number of major crops that are important for human and animal nutrition including cassava, different cereals and forages as well as other important sources for commercial products such as oil, fiber, and sugar (e.g. sugar beets and most forage grasses). The majority of current breeding programs for allogamous crops require more than a decade to develop a new cultivar as they are based on phenotypic recurrent selection (Vogel and Pedersen, 1993; Casler and Brummer, 2008). For this reason, genetic gain using conventional phenotypic programs tends to be low and, for example, has not exceeded 1.3% per year in several forage species (e.g. Gates et al., 1999; Humphreys, 1999; Wilkins and Humphreys, 2003; Casler, 2012).

The development of cost-effective and high-throughput genotyping methods have made it possible to improve selection efficiency through genomic selection (GS) (Varshney et al., 2014). GS, first proposed by Meuwissen et al. (2001), makes use of phenotypic and genotypic records collected on a set of individuals, called the training or reference population, to predict the performance of other individuals with genotypic records only. These effects can be used to predict the potential of genotyped individuals with no phenotypic records in the training populations. GS is now a demonstrated method to improve selection efficiency for different animals such as dairy cattle (Hayes et al., 2009; García-Ruiz et al., 2016); trees such as apples (Muranty et al., 2015); forage crops such as ryegrass (Pembleton et al., 2018); and cereal crops such as bread wheat (Daetwyler et al., 2014) and maize (Krchov and Bernardo, 2015). The main advantages of applying GS on crops includes reducing the length of the breeding cycle (i.e. allowing a selection of superior plants at the seedling developmental stage), which can improve genetic gain per unit of time (Lin et al., 2016); whilst reducing the cost per unit of genetic gain (Lin et al., 2017a).

Combining GS with other modern breeding strategies may further enhance its efficiency on improving genetic gain. For instance, Cabrera‐Bosquet et al. (2012) recommended combining GS with high-throughput phenotyping methods to further increase the efficiency of both methods. Doubled haploid technology, the doubling the chromosome number of a haploid cell using colchicine, is used in several species. Extensions to GS that focused on selecting the best haplotypes (Optimal Haploid Value Selection) has been proposed to increase genetic diversity and genetic gain in crops (Daetwyler et al., 2015).

The recent development of “speed breeding” (SB) protocols has the potential to significantly accelerate breeding programs for different crops by reducing the generation time (Watson et al., 2018; Voss-Fels et al., 2018). In SB, plants are grown in controlled environments with continuous light for 22 h per day at optimal temperature. The advantage of SB has been proven for many crops such as *Brassica* species, bread wheat, durum wheat, barley, chickpea, pea, grass pea, quinoa, oat, *Brachypodium distachyon,* and peanut; and at least four generations have been achieved in a single year using SB (O'Connor et al., 2013; Ghosh et al., 2018; Watson et al., 2018). Thus, combining GS and SB should allow for more intense and more frequent selection stages and contribute to higher genetic gain per year. Although SB has already been used in practice (Hickey et al., 2017; Gorjanc et al., 2018), such novel breeding programs need to be adequately tested for optimal utilization in a commercial setting.

While breeding strategies such as GS and SB show considerable promise, their efficient implementation requires optimization as implementation could be achieved in many ways. Large scale empirical testing of many scenarios is costly and slow. Alternatively, computer simulation can be a time- and cost-effective tool to predict the outcome of multiple breeding designs and strategies. Simulation can facilitate the assessment of optimal population sizes, selection intensities or other breeding program parameters ending with a small number of recommended schemes for the industry. Planning a simulation study for a breeding program requires 1) information about the genomic characteristics of the empirical breeding population for the target species; 2) understanding the genetic architecture and variance components of the traits under investigation; 3) knowledge about current breeding practices; and 4) designing and integrating new strategies into the current breeding practices (e.g. Lin et al., 2016). Once several alternate breeding programs have been simulated, the most effective in terms of additional genetic gain and cost can become the focus for empirical testing.

A previous study simulated a simple breeding program which combined both SB and GS (SpeedGS) in wheat (Voss-Fels et al., 2018). However, they used 1,020 loci and they assumed that they are all causative variants. In reality, the causal variants are unknown and the genomic prediction models depend on linkage disequilibrium between causal and genotyped loci (Meuwissen et al., 2001). Simulation studies showed that including causative variants in the prediction model result in higher prediction accuracy, which contributes to overestimating the potential of breeding schemes that simulate only causal variants (Meuwissen and Goddard, 2010). For this reason, simulated causal variants should be masked in the GS analysis to avoid overestimating the prediction accuracy.

In this study, through stochastic computer simulation, the potential of incorporating both GS and SB strategies into allogamous crop breeding programs was investigated to improve multiple traits with different genetic architectures. The main strategy involved selecting parents for crosses based on their genomic estimated breeding values (GEBVs), generating their progeny through SB, and repeating the process multiple times in a single year. Multiple designs were tested omitting some of the conventional phenotypic selection (PS) stages to study their effects on the genetic gain, GS accuracy, and inbreeding rate. We aimed to compare the genetic gain per breeding cycle, genetic gain per year, accuracy of GS, and inbreeding levels for each proposed scheme. In each scheme, we simulated one, two, or three SB rounds and we also aimed to study the effect of reducing the number of full-sib plants in the initial population. As a case study, the commercial breeding program for the pasture crop tall fescue (*Fescue arundinacea*; 2n = 6x = 42; G1G1G2G2PP) was simulated. Tall fescue is a hexaploid pasture crop that evolved from the hybridization of three diploid genomes, G1, G2, and P. To the best of our knowledge, this is the first simulation study to investigate the potential of breeding programs for allogamous crops that combine GS and SB and its effect on inbreeding.

## Materials and Methods

**Figure 1:**
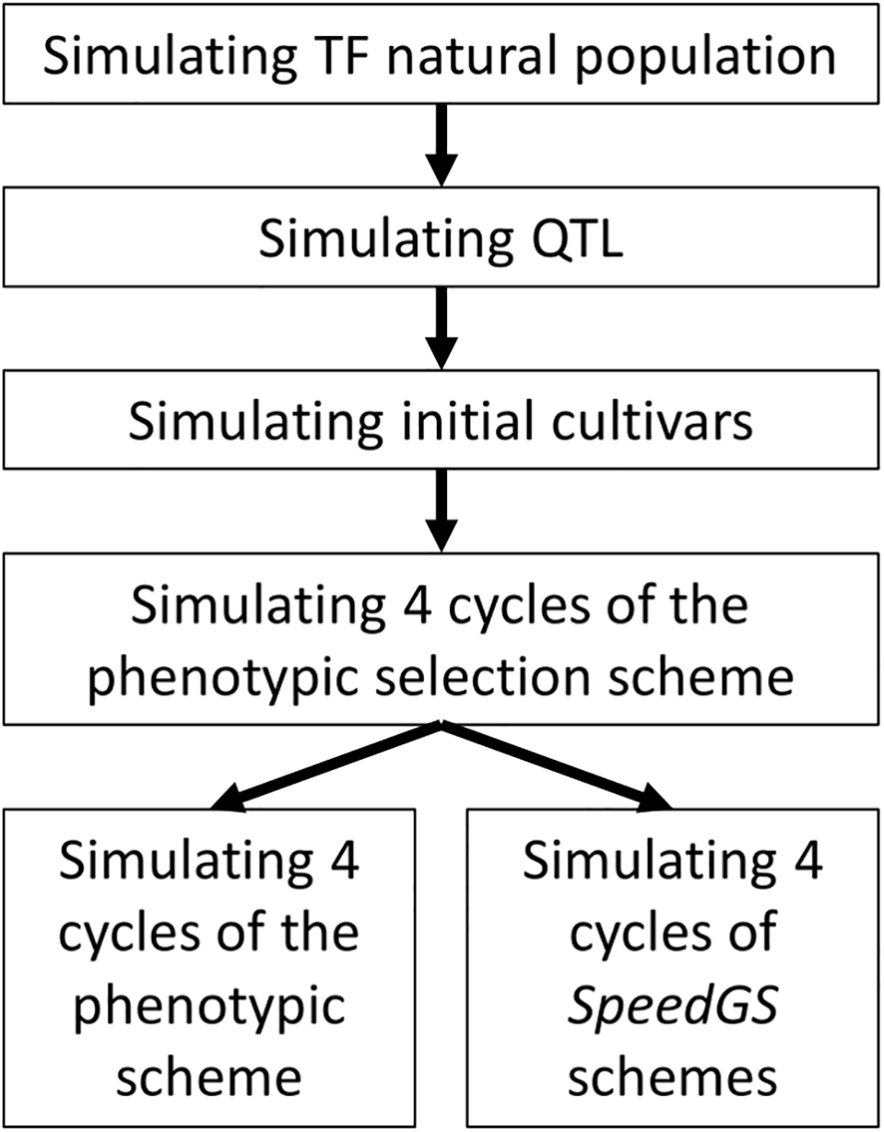
General diagram for simulated breeding program. TF, Tall fescue.

The simulated phenotypic scheme is similar to many worldwide commercial programs for allogamous crops. The simulation was conducted in the following steps (**Figure 1**). First, 2.1 million equally distributed loci were simulated on 21 chromosomes, representing a base population that mimics genomic characteristics of a tall fescue natural population (including the extent of linkage disequilibrium, LD, and heterozygosity, *He*; Brazauskas et al., 2011; Fiil et al., 2011). Second, 1,000 quantitative trait loci (QTL) per trait (five traits) were randomly sampled from the simulated 2.1 million loci. The effect of these QTL was sampled from different distributions to match the proposed architecture for each trait. Third, 20 initial breeding cultivars were simulated from the base population to match the diversity of commercial tall fescue cultivars. Finally, conventional PS and our proposed SpeedGS schemes (breeding program which combined both speed breeding and genomic selection) were simulated from the initial cultivars. Each step is described in detail in the following sections. One hundred replicates of breeding programs with different schemes were simulated.

### Simulating Tall Fescue Base Population and Cultivars

The first step in the present study is to develop a simulated population with similar genomic characteristics to the natural population of the target crop, tall fescue in our case. As tall fescue is an allohexaploid crop, we used the software PolySim (Jighly et al., 2018b) to simulate the evolution of the hexaploid crop from its diploid progenitor species. The parameter file used for PolySim can be found in **supplementary text 1**. We started the simulation with a common diploid ancestor that underwent three speciation events to form the three diploid ancestors of tall fescue at generations 8,000, 8,200 and 15,000 (**Figure S1**). The first two diploids were hybridized to form the tetraploid ancestor *Fescue glaucescens* (G1G1G2G2) at generation 16,000. The hexaploid tall fescue was evolved at generation 25,000 by hybridizing the tetraploid and the third diploid (*Fescue Pratensis*; PP). We stopped the simulation at generation 105 to ensure that the hexaploid populations reached mutation-drift equilibrium. For all newly evolved taxa, populations were expanded exponentially within 100 generations to reach the final population size. The number of generations in each stage was selected to run the analysis long enough to reach mutation-drift equilibrium (Jighly et al., 2018b).

The hexaploid population was simulated with 10^4^ individuals, seven haploid chromosomes, and 10^5^ loci per chromosome. The mutation rate was set to 10^-5^ per locus and recombination were sampled from Poisson distribution with *λ* equal to one crossover per chromosome, where each of 21 chromosomes was 100 cM. Because tall fescue is an allohexaploid, we treated it as a diploid organism having 21 independent diploid chromosomes (Jighly et al., 2018b). Twenty cultivars were simulated by randomly selecting 1,000 plants from the base population and running the simulation for an extra 100 generations with no mutation. These cultivars were used as the founders for the breeding program. All these parameters were selected following Lin et al. (2016) as tall fescue has similar or more extensive LD compared to ryegrass (Forster et al., 2014) and because the main aim of the present study is to demonstrate the potential of implementing SpeedGS in breeding programs.

### Simulating Traits and QTL Effects

We simulated five different traits to be subject to selection which were heading date (HD) with narrow sense heritability *h* *^2^* = 0.7, forage yield (FY) *h* *^2^* = 0.3, persistency (Per) *h* *^2^* = 0.1, seed yield (SY) *h* *^2^* = 0.4, and quality (Q) *h* *^2^* = 0.3 (**Table 1**). FY was simulated to have correlation with SY (r = 0.2) and HD (r = 0.3). These values were selected based on results from (Nguyen and Sleper, 1983; Araujo et al., 1983; Veronesi and Falcinelli, 1988; Ebrahimiyan et al., 2013a; Ebrahimiyan et al., 2013b) and knowledge from other similar pasture species (unpublished data). Each trait had 1.000 different additive QTL that were randomly sampled from loci with minor allele frequency >5% (Lin et al., 2016). To simulate pleiotropy and to achieve the genetic correlations, 500 QTL were shared among FY, SY. and HD (**Table 1**).

**Table 1:** Information about simulated traits: narrow sense heritability on diagonal with selection weight in the SpeedGS stage between brackets, correlation between traits below diagonal and number of shared QTL above diagonal.

Trait	FY	HD	SY	Q	Per
FY	0.3(0.25)	500	500	0	0
HD	0.3	0.7(0.1)	500	0	0
SY	0.2	0	0.4(0.15)	0	0
Q	0	0	0	0.3(0.15)	0
Per	0	0	0	0	0.1(0.35)

FY, forage yield; Per, persistency; SY, seed yield; Q, quality; HD, heading date.

QTL effects for FY, Per. and SY were simulated from the normal distribution N ∼ (0, 1), while QTL effects of Q and HD were simulated from two different normal distributions. It is known that HD has some QTL of large effect (Barre et al., 2009; Fè et al., 2015) and this is to some extent also true for Q. Hence, we wanted to allow for large and small effect QTL for both traits and, thus, standard methods to simulate correlated QTL (e.g. the R function mvrnorm in MASS package) are not applicable to this case and we had to develop a new method for this purpose. Unfortunately, HD architecture is unknown for tall fescue, but one could expect that it is comparable to perennial ryegrass. Fè et al. (2015) detected 14 QTL that combined explain approximately 31% of HD narrow sense heritability in perennial ryegrass. Given that associated SNPs are expected to explain less additive variance than causal variants depending on the level of LD (Purcell et al., 2003) and some homoeologous genes can work in duplicates or triplicates in polyploids (Takahagi et al., 2018), we proposed that 20 QTL for HD could explain 50% of the total trait genetic variance. Similarly, we assumed that 100 QTL for Q could explain 50% of the total additive variance. Thus, the remaining small effect QTL (980 for HD and 900 for Q) were simulated from normal distribution N ∼ (0, 1), while the large effect QTL were simulated from normal distribution with a scaled standard deviation N ∼ (0, *m*) in which:

$$m=\frac{e\;(n-1)}{l\;(1-e)}$$

Where e is the total genetic variance explained by large effect QTL (ranged between 0 and 1); n is the total number of simulated QTL and *l* is the number of large effect QTL.

### Simulating Correlation Between QTL Effects

Simulating correlated traits is usually done by multiplying a matrix *R* (*n* × *p*) of random QTL effects with the Cholesky decomposition of the targeted (*p* × *p*) correlation matrix *C* (n QTL for p traits); assuming C is a Hermitian, positive definite matrix. This works because Var(*R*) = *I*; in which *I* is the identity matrix. However, if the QTL effects for multiple traits were sampled from multiple normal distributions with different variances (such as our case), the variance of *R* will not be equal to that of the identity matrix. For this reason, we proposed the below method to solve this problem.

First, simulate an (*n* × *p*) matrix (*R*) in which random QTL effects for each trait were drawn from different normal distributions, i.e. Var(*R*) ≠ *I*. Second, rescale *R* to keep its variance equal to the identity matrix variance by multiplying it with the inverse of a matrix *L*, the Cholesky decomposition of the variance of *R* (Var(*R*) = *L* × *L* *^T^*); where *L* *^T^* is the transpose of *L*.

$$RS=R\times L^{-1}$$

*RS* can be then multiplied with *D*, the Cholesky decomposition of the required correlation matrix *C* (*C* = *D* × *D* *^T^*); where *D* *^T^* is the transpose of *D*; to get the final correlated QTL effects, where 20 QTL explain 50% of the genetic variance and 980 QTL explain the other 50% for HD.

### Simulating True Breeding Values and Phenotypes

True breeding values (TBVs) for individuals were calculated as the sum of all QTL effects multiplied by their allelic dosage (recorded as 0, 1. or 2). Phenotypes were simulated considering the *h* *^2^* for each trait by adding random environmental error terms to TBVs as Pheno=TBV+*e*; where *e* is a random normal deviate sampled from $N∼(0,\;\sigma_{e}^{2})$ and errors for different traits were independent. The error variances were rescaled after each breeding cycle to ensure constant *h* *^2^* during the breeding program.

### Simulating the Phenotypic Breeding Program

**Figure 2:**
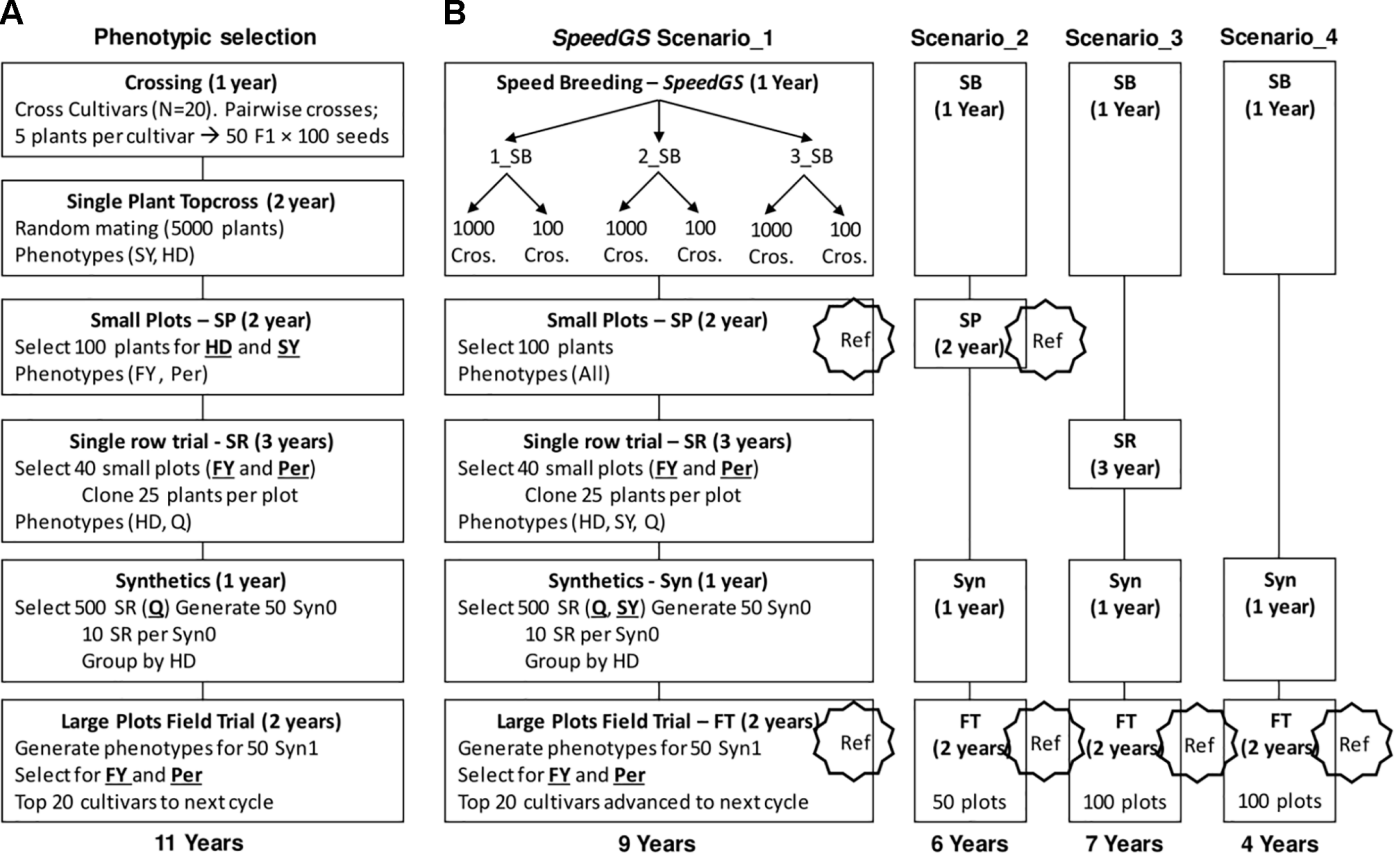
Detailed stages of **(A)** the phenotypic and **(B)** the proposed SpeedGS scenarios.

We ran eight cycles of the following PS program. The PS program required a total of 11 years to release new cultivars with six major stages of which four containing a selection step (**Figure 2A**). The first stage (crossing; one year) of the program started with selecting five random plants from each of 20 cultivars. Then, randomly crossing the selected plants from each pair of cultivars to generate a total of 50 F1 populations with 100 seeds each (five F1 populations from each pair of cultivars × 10 pairwise cultivar crosses). In stage two (top cross; two years), the resulting 5,000 seeds would be planted in a single-spaced plant trial with random mating among them. At this stage, selection for HD and SY was applied by first discarding the earliest 1,000 plants (20%) for HD and then randomly selecting 100 plants from the top remaining 3,000 plants (60%) for SY.

Five hundred seeds from each of the 100 selected plants were planted in small plots (stage three; two years) and these small plots were phenotyped for FY and Per for selection. Equal selection pressure was applied, and phenotypes were standardized. The top 40 small plots were selected based on FY and Per and 25 plants from the top 80% plants for FY and Per within each small plot were cloned to be planted in a single row trial. This resulted in 1,000 plants (25 plants × 40 small plots) being grown in single rows and phenotyped for HD and Q, which is stage four (single rows; three years). In stage five (synthetics; one year), the top 500 rows for Q were selected and were grouped into 50 synthetic populations where each involved 10 rows with similar HD phenotypes. In the final stage (field trial; two years), the 50 synthetic populations were planted in large plots and the top 20 large plots were selected as cultivars for the next breeding cycle based on their FY and Per performance. Again, both FY and Per were selected for simultaneously using an index with an equal 50% weight for each trait.

### Simulating Different Combined Speed Breeding and Genomic Selection (SpeedGS) Schemes

In all proposed SpeedGS schemes, we replaced the first two stages in the PS scheme with one year of SB, with one to three rounds (**Figure 2B**). Having one SB round indicates a scheme that utilizes GS only without SB. For each SB round within this year, we genotyped 1,000 single plants, predicted their GEBVs and selected 1,000 × 1 progeny or 100 × 10 progenies diverse crosses for the next SB round or the next stage of the breeding program. We simulated genomic parent average GEBVs for all possible crosses (1,000×999/2=499,500 crosses) and randomly selected the best crosses from the top 10,000 possible combination of parents. All traits were selected simultaneously with index weights of 0.35, 0.25, 0.15, 0.15, and 0.1 on the GEBVs of Per, FY, SY, Q, and HD; respectively. The weights were selected to give higher intensities for traits with lower heritabilities with more emphasis on FY and were combined as *Index* = Σ *b*_i_ *X*_i_, where *b* *_i_* is the index weight and *X* *_i_* is the plant GEBV for trait *i*. The cultivars released after the fourth PS program were used to commence the first SpeedGS cycle, while the small plots from the first four phenotypic cycles (400 small plots) were genotyped and used as a reference population to train the GS prediction equation for all five traits. The genotyping for small plots were recoded as the allelic dosage of SNPs (2 × the allele frequency) calculated using 20 randomly selected plants per plot (Ashraf et al., 2014; Lin et al., 2016). Four breeding program cycles were run for all SpeedGS scenarios.

We proposed four major SpeedGS schemes (**Figure 2B**). For each major scenario, we tested whether it is best to have one, two, or three SB rounds or whether it is best to select 1,000 crosses × one offspring, or 100 crosses × 10 offspring during the SpeedGS stage. For this reason, the total number of tested scenarios was 24 (six scenarios per each of the four major SpeedGS schemes; **Figure 2B**).

- Scenario one (requiring 9 years) involved the same stages as the phenotypic program except for replacing the first two conventional stages with the 1-year SpeedGS stage
- Scenario two (6 years) omitted the single row stage
- Scenario three (7 years) left out the small plots stage
- Scenario four (4 years) omitted both the small plots and the single row stages so it had the shortest cycle.

The initial population (Topcross, **Figure 2A**) in the PS program consisted of 5,000 individuals, while the initial population for the proposed SpeedGS programs contained 1,000 individuals to reduce genotyping cost. For the first SpeedGS cycle, 1,000 individuals were randomly selected from the 5,000 individuals of the fifth phenotypic cycle initial population. small plots and field trial stages were genotyped and added to the reference population to update the GS prediction equation after every cycle. Field trial size was changed to 100 instead of 50 for the third and the forth scenarios to partially compensate for omitting small plots. After the single row trial, selection was conducted on SY and Q (equal index) instead of Q only. Everything else was the same as the PS program.

As SpeedGS involving multiple selection stages in a short time period, we tried to investigate the components that affect inbreeding, and to find possible ways to reduce inbreeding. We considered the fourth major scenario with three SB rounds and 1,000 crosses during the SpeedGS as our base for comparison. We investigated the following alternative scenarios in our study: 1) developing the synthetic cultivars from five plants instead of ten; and 2) developing the initial population starting from cycle 5 from 100, 200 or 1,000 crosses instead of 50 crosses × 100 plants. We kept the same pairwise crossing scheme but involved more plants per cultivar to be crossed. In other words, we aimed to study the effect of reducing the number of full-sib plants in the initial population.

### Statistical Analysis

We tracked the linkage disequilibrium (LD) and heterozygosity (*He*) of the hexaploid population after its first appearance every 5,000 generations to ensure that the population reached equilibrium between mutation and drift and to be compared with empirical values and expected values given the simulation parameters. However, given the limited information available on tall fescue, we compared it with ryegrass as it is expected to have similar LD with tall fescue (Forster et al., 2014). LD was calculated following Hill and Weir (1988) between each pair of loci with minor allele frequency (MAF) > 5% within the same chromosome. The expected LD decay was calculated following Tenesa et al. (2007) method which considering the relation between LD and the effective population size (*N* *_e_*) considering the mutation rate as [E(*r* *^2^*) = 1/(2+4*N* *_e_* *C*) + 1/*n*]; in which *C* is the genetic distance in Morgan and n is the sample size. The expected *He* was calculated following Crow and Kimura (1970) method taking into account the presence of only two alleles per loci as: [E(*He*) = 4*N* *_e_* *µ*/(8*N* *_e_* *µ*+1)]; in which *µ*is the mutation rate. Two-sample student’s t tests were performed to investigate the level of significant differences between scenarios or different analyses, where required. Throughout the manuscript, comparisons with p<0.01 were declared to have significant differences.

To estimate SNP effects, we randomly selected 100,000 SNPs from the simulated 2.1 m loci after excluding the causal variants or the simulated QTL. These loci had MAF > 5% in the base population so it is possible for some of them to get fixed before the commencement or during the SpeedGS program. SNP effects were estimated using the Bayesian ridge regression (BRR) method implemented in the R package Bayesian Generalized Linear Regression, BGLR (Pérez and de Los Campos, 2014). BRR assumes that regression coefficients have common variances so all SNPs with similar allele frequencies should have similar contribution to the additive variance (Gianola, 2013). We ran each BGLR analysis for 50,000 iterations and discarded the first 10,000 as burn in. GEBVs were calculated as $GEBV=Z\hat{\beta }$ where *Z* is a matrix of plant SNP allelic dosages and $\hat{\beta }$ is a vector of estimated SNP effects. The accuracy of GS was investigated for all scenarios and all three SB rounds to allow for comparing GS accuracy with SB rounds = 1, 2, or 3. The accuracy was calculated as the Pearson correlation between GEBVs and TBVs. The initial population for the fifth phenotypic cycle was used as the base to compare its genetic gain with the following cycles as a standardized difference in average TBVs using the following equation:

$$\Delta \sigma_{g}=\frac{av.(TBV_{c_{i}})-av.(TBV_{c_{5}})}{std.(TBV_{c_{5}})}$$

where av. (TBV_c5_) and av.(TBV*ci*) are the average TBVs of the initial population in cycle five and cycle *i* (*i* ranged from six to nine), and std.(TBV*c5*) is the TBV standard deviation in the fifth cycle initial population. In cycle nine, we only generated the initial population to track genetic gain.

The initial populations of breeding cycles five to nine were also used to track the increase in the inbreeding coefficient (*F*). The genomic relatedness matrices (GRM) for initial populations were generated following the first method described in VanRaden (2008) and used the allele frequencies observed in the initial population of the phenotypic cycle five. We calculated the mean population F for each cycle as the average initial population GRM diagonal elements minus one. The rate of inbreeding change (*ΔF*) between neighboring cycles (cycle *c* and *c-1*) was calculated according to Falconer and Mackay (1996) as:

$$\Delta F=\frac{F_{c}-F_{c-1}}{1-F_{c-1}}$$

## Results

### Genetic Gain Per Breeding Cycle

**Figure 3:**
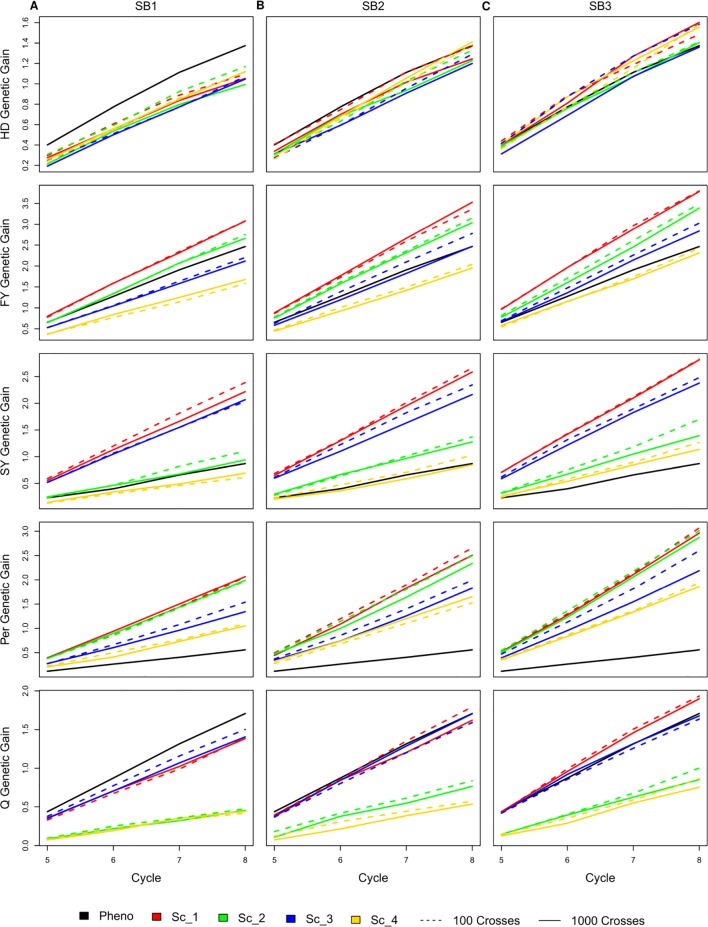
Cumulative genetic gain across cycles for all simulated breeding schemes for all traits with SB equal to **(A)** one, **(B)** two, and **(C)** three.

Selecting 100 instead of 1,000 crosses during the SpeedGS stage resulted in no significant differences in genetic gain for all traits and under all scenarios. Generally, the first scenario performed better than all other scenarios for all traits after four breeding cycles. For each of the four major scenarios suggested in this study, we tested the case of having one crossing round, which tested implementation of GS without applying SB. For all traits in all scenarios, having more SB rounds generally resulted in significantly (SB1 vs SB3) higher genetic gain per cycle (**Figure 3**; **Table S1**). Compared to PS, having three SB rounds resulted in similar or significantly higher genetic gain per cycle, except for Q in scenarios two and four (**Figure 3**).

The genetic gain of all four SpeedGS scenarios were comparable after each SB round for HD. PS performed significantly better than all four scenarios for HD when considering SB1, but was comparable to SB2 (PS slightly higher) and SB3 (PS slightly lower). For FY, scenario one performed better than all other scenarios followed by scenarios two and three. Scenarios one and two consistently showed significantly higher genetic gain compared to scenario four, while their differences from scenario three were not significant except between scenario one and three for SB3. The PS exhibited significantly higher genetic gain than scenario four for SB1 and significantly lower genetic gain than scenario one for SB2 as well as scenarios one and two for SB3. Scenarios one and three showed significantly higher genetic gain for SY compared to scenarios two and four as well as the PS for all SB rounds. SY genetic gain for scenario two was significantly higher than the PS only for SB3.

All SpeedGS scenarios, regardless of the number of SB rounds or the crossing scheme, resulted in significantly higher genetic gain compared to PS for Per, which had the lowest heritability. Scenarios one and two steadily showed significantly higher genetic gain compared to scenarios three and four. For the trait Q, PS always showed higher genetic gain than scenarios two and four but resulted in no significant differences between scenarios one and three, involving the single row planting stage. Scenarios one and three were not significantlydifferent, regardless of the number of SB rounds, and this was also the case for scenarios two and four. However, scenarios one and three showed significantly higher genetic gain compared to scenarios two and four.

### Genetic Gain Per Year

**Figure 4:**
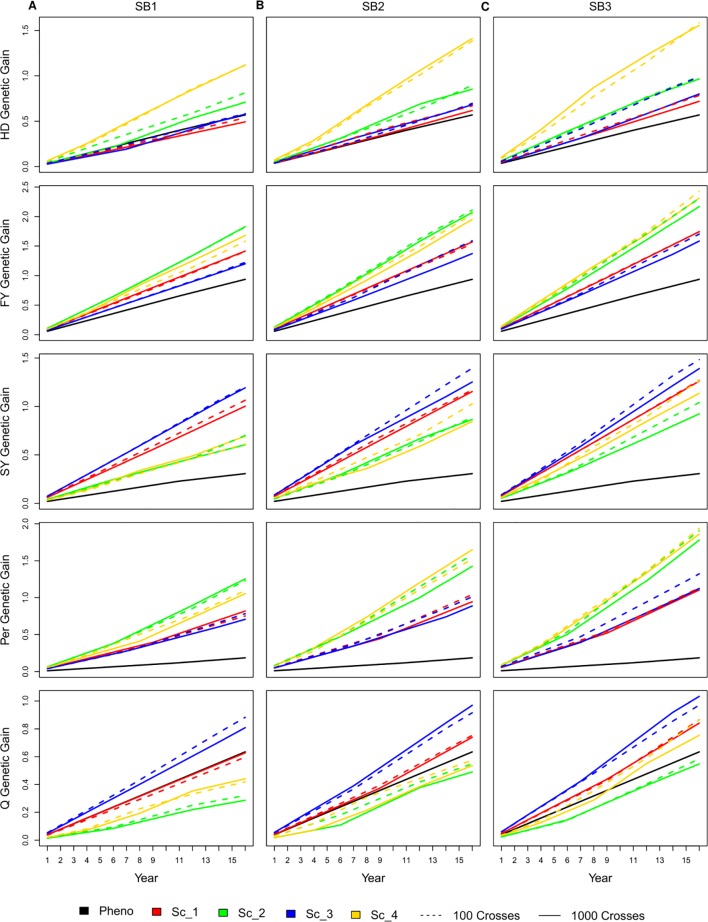
Cumulative genetic gain across year for all simulated breeding schemes for all traits with SB equal to **(A)** one, **(B)** two, and **(C)** three.

Similar to the genetic gain per cycle, having 100 or 1,000 crosses during the SpeedGS stage resulted in no significant differences in genetic gain per unit of time (year), regardless of the scenario or the number of SB rounds. As SpeedGS schemes had a shorter cycle, the general trend showed that they performed better than the PS in most cases especially after three SB rounds (**Figure 4**). For example, the genetic gain of scenario four with three SB rounds was 2.7, 2.5, 3.7, 10.0, and 1.2 times higher than PS for HD, FY, SY, Per, and Q after 16 years; respectively (dividing the genetic gain of all traits in scenario four on the genetic gain of the PS; inferred from **Figure 4**).

For HD, shorter scenarios (two and four) always exhibited significantly higher genetic gain except for scenario two with one SB round compared to PS. The genetic gain of the longer scenarios (one and three) was always comparable to PS, although they were slightly higher with three SB rounds. The shortest scenario (four), resulted in significantly higher genetic gain compared to all other SpeedGS scenarios (**Figure 4**). For FY, the genetic gain in all scenarios showed significantly higher genetic gain compared to PS except for scenario three when running a single SB round. Scenarios two and four consistently displayed higher genetic gain compared to scenarios one and three and the differences were significant for SB2 and SB3. PS for SY resulted in a significantly lower genetic gain compared to all other scenarios regardless the number of SB rounds. The longer scenarios, one and three, showed significantly higher genetic gain compared to the shorter scenarios when running one or two SB rounds. When running three SB rounds, the differences were significantly higher only when compared with scenario two. Similar to SY, all scenarios showed significantly higher genetic gain than the PS for Per. The shorter scenarios, two and four, always showed significantly higher genetic gain compared to the longer scenarios except when comparing scenario four with scenarios one and three for SB1. Q was the only trait which PS exceeded some SpeedGS scenarios (**Figure 4**). PS significantly exceeded scenarios two and four when running a single SB round. On the other hand, PS showed significantly lower genetic gain compared to scenario three for all SB cases as well as scenario one for SB3.

### Accuracy of Genomic Selection

**Figure 5:**
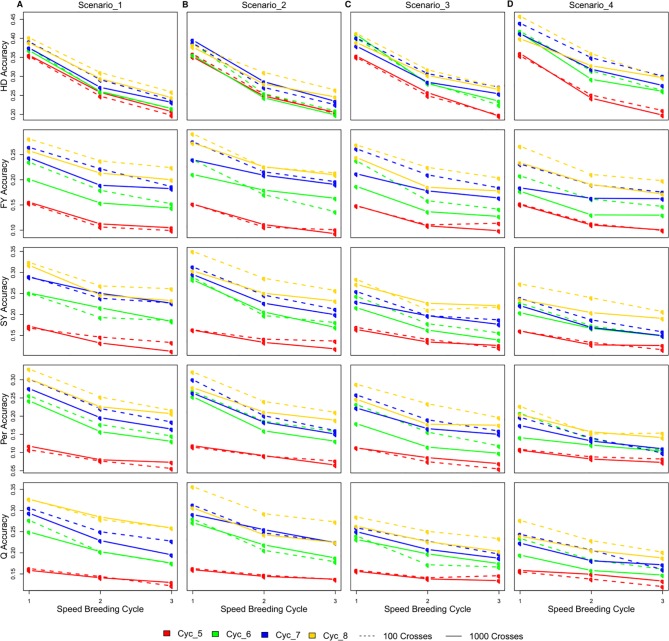
The changes in the accuracy of genomic selection after each of the three SB rounds in SpeedGS scenarios **(A)** one, **(B)** two, **(C)** three, and **(D)** four.

The accuracy of GEBVs varied considerably among different SpeedGS scenarios, SB rounds within the SpeedGS stage, and different breeding cycles (**Figure 5**; **Table S2**). Scenarios with 100 crosses × 10 plants during the SpeedGS stage improved accuracy over 1,000 crosses × 1 plant (**Figure 5**). Prediction accuracy decreased with each additional SB round. However, this decrease was not linear with a larger difference between SB1 and SB2 compared to the difference between SB2 and SB3. For this reason, we run a single breeding cycle of the SpeedGS scenario four with six SB rounds. The results suggested reaching a stable value for accuracy after the fourth SB round for all traits (**Figure S3**). Accuracy increased as overall breeding cycles increased due to expanding reference populations. The first SpeedGS cycle (cycle five) resulted in very similar accuracies in all scenarios. In the following cycles (cycle six to eight), the highest accuracy was observed in the second scenario followed by the first for all traits except for HD. The last scenario, which had the shortest time, had the highest accuracy for HD, but the lowest accuracy for the remaining traits. HD was predicted with high accuracy compared to other traits, while Per showed the lowest accuracy.

### Inbreeding

**Figure 6:**
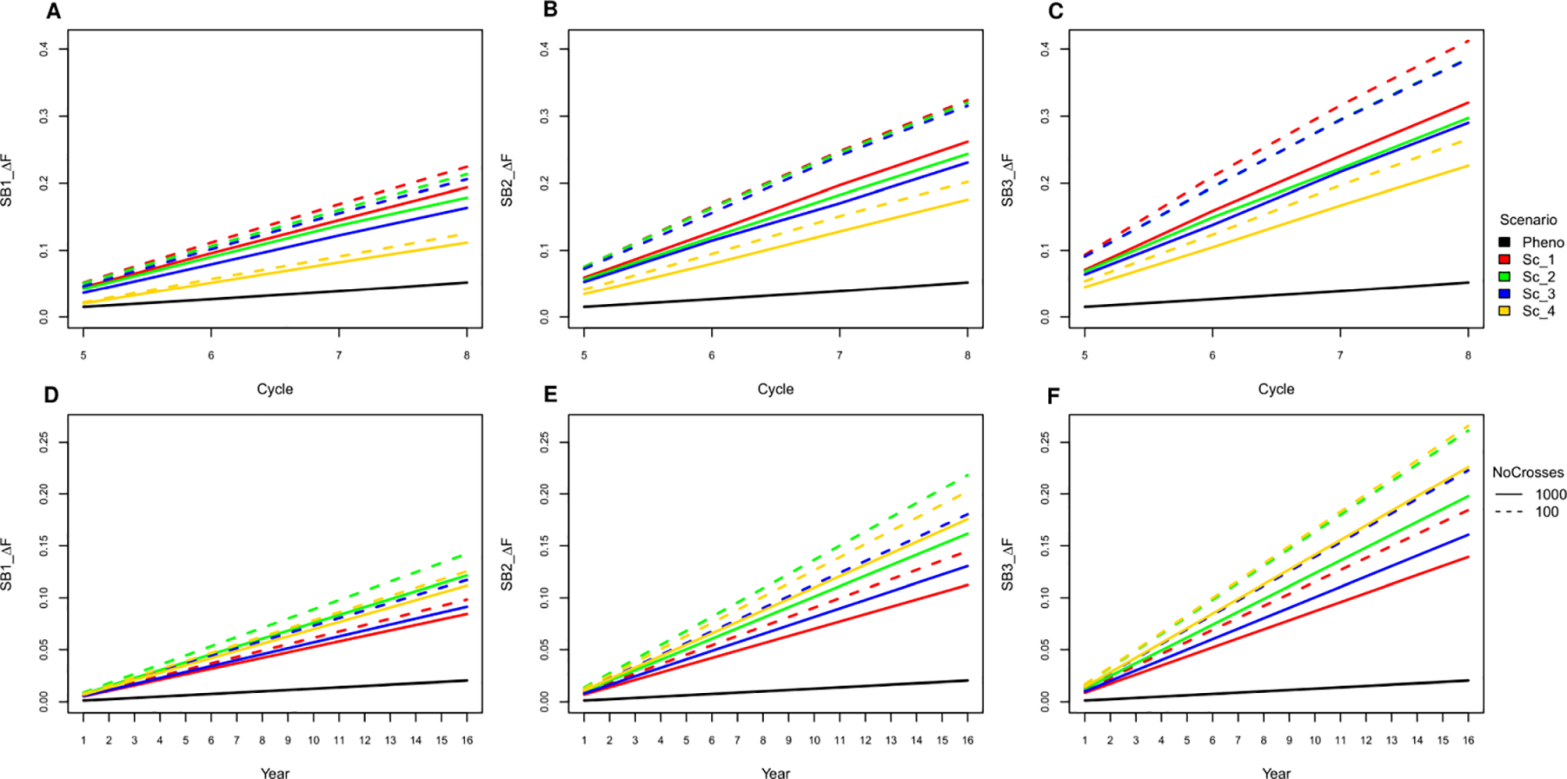
Cumulative inbreeding rate increase across cycle **(A**−**C)** and per year **(D**−**F)** for SB equal to one, two and three; respectively.

All SpeedGS scenarios resulted in larger increases in inbreeding per cycle and reductions in genetic diversity compared to PS even when only one SB round was applied. Extra SB rounds resulted in higher inbreeding rate (**Figure 6**; **Table S3**) and lower diversity (**Table S4**). For example, applying three SB rounds when running 1,000 SpeedGS crosses for the fourth scenario resulted in twice the inbreeding rate (0.22) obtained when applying a single SB round only (0.11). The first scenario resulted in the highest inbreeding rate per cycle followed by the second, while the fourth had the lowest inbreeding rate. However, when considering the inbreeding per year, the fourth scenario had the highest inbreeding rate, while the first resulted in the lowest. Having 100 crosses × 10 plants during the SpeedGS stage resulted in larger increase in inbreeding.

**Figure 7:**
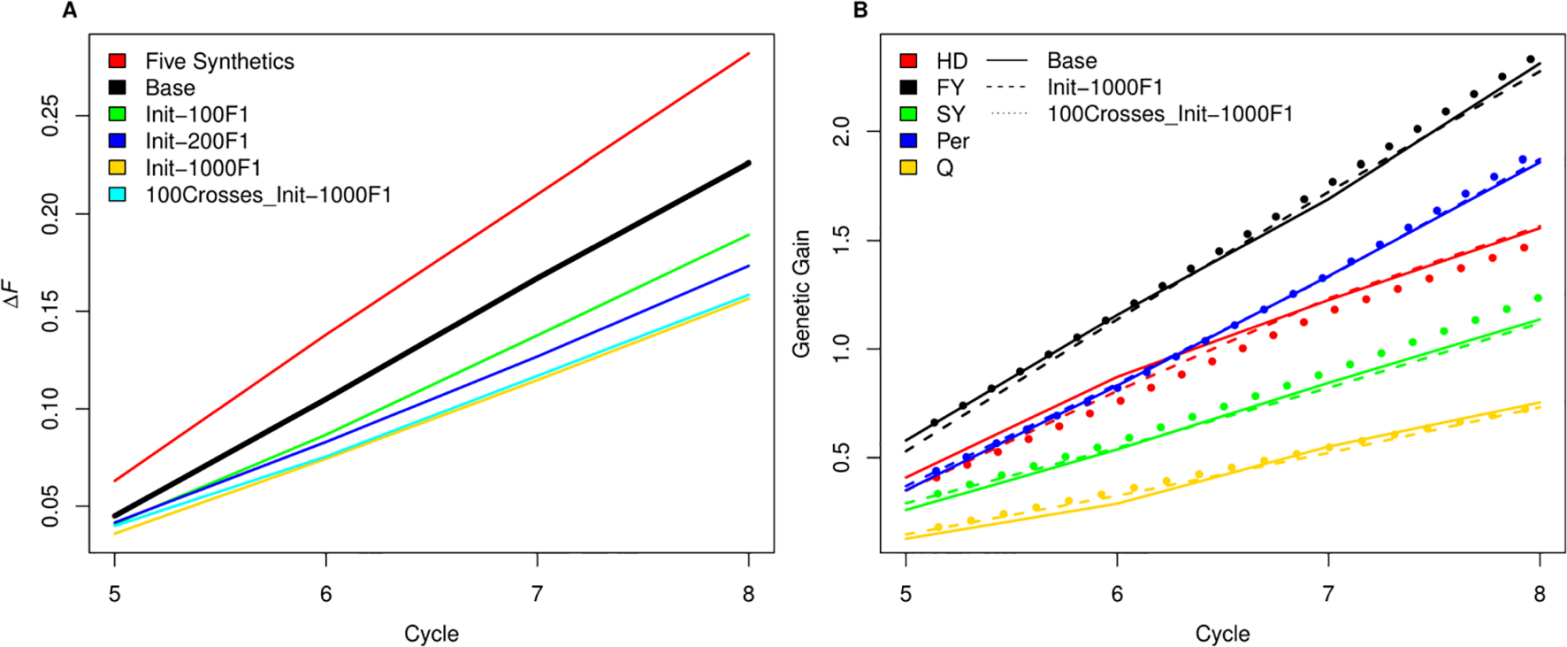
**(A)** A comparison between inbreeding rate for scenario four with three SB rounds and 1000 crosses during SpeedGS stage (the Base scenario in the black line) with alternative scenarios of having five plants per synthetic instead of 10 (red line), or developing the initial population from 100 F1 × 10 progenies (green), 200 F1 × five progenies (blue) or 1000 F1 × one progeny with 1000 crosses during SpeedGS (orange) or 100 crosses (cyan). All other parameters were similar for all lines. **(B)** A comparison for genetic gain for all traits among the base scenario (straight line) and the alternative scenarios developed from 1000 F1 with 1000 crosses × one progeny during SpeedGS (dashed line) or 100 crosses × 10 progenies (dotted line).

As SpeedGS schemes resulted in higher inbreeding rates, we tried to investigate possible modifications to the proposed SpeedGS programs that can limit this sharp increase (**Figure 7A**). We considered the fourth scenario with 1,000 crosses as our base scenario to compare the changes. Our results showed that having a larger number and more diverse plants to form the synthetic populations could affect the inbreeding rate as using 10 plants instead of five significantly reduced the inbreeding rate. Generating the initial population using much larger number of crosses resulted in the largest reduction of inbreeding rate and the larger the number of crosses, the lower the inbreeding rate. Developing the initial population of 1,000 individuals using 1,000 random crosses decreased the inbreeding rate by 30% from 0.226 to 0.158 compared to the base scenario. Interestingly, the same inbreeding level was obtained whether we used 100 or 1,000 crosses during the SpeedGS stage (**Figure 7A**), without affecting genetic gain (**Figure 7B**).

## Discussion

The revolution of next generation sequencing methods has moved genomic selection from a theoretical possibility to a practical choice for modern commercial breeding programs. Several empirical (e.g. Hayes et al., 2009; Daetwyler et al., 2014; Krchov and Bernardo, 2015; Muranty et al., 2015; Pembleton et al., 2018) and simulation-based studies (e.g. Yabe et al., 2013; Lin et al., 2016) have demonstrated the importance of adapting GS into current practices in both plant and animal breeding. Today, after almost two decades since the inception of the GS idea (Meuwissen et al., 2001), the research question has changed from whether using GS will increase genetic gain and profits, to how one can further increase the efficiency of GS. In this paper, we investigated the pros and cons of combining the newly proposed breeding strategy “speed breeding” with GS in allogamous crop breeding programs. Generally, we found that breeding schemes that involved more SB rounds have significantly higher genetic gains for different traits compared to schemes that used GS only, and they both (GS and SpeedGS schemes) outperformed conventional PS in almost all scenarios and traits. However, we found that the more SB rounds were performed in each breeding cycle the faster the shrinkage in the genetic diversity of the breeding population, which may limit the long-term genetic gain (Goddard, 2009). For example, the inbreeding level was doubled when having three SB cycles compared to the scenario with only one SB cycle.

### Simulating the Tall Fescue Base Population

The simulated population using PolySim followed the theoretical expectation for LD and *He*. Our population had very similar LD decay pattern to the expected decay considering our simulation parameters (**Figure S2**). Previous empirical studies on ryegrass also reported similar quick LD decay in less than one centimorgan (Brazauskas et al., 2011; Fiil et al., 2011). *He* was equal to 0.218±0.01, which is comparable to the expected *He* value of 0.222 as well as the empirical *He* reported for perennial ryegrass in Fiil et al. (2011), which was 25.9%. The average *He* in Brazauskas et al. (2011) was equal to 0.4 but they used microsatellite markers, which are known to be highly mutable and have multiple alleles per loci which contribute to higher *He* (Ellegren, 2004).

### Genetic Gain Per Cycle

Considering cumulative gain across cycles, increasing the number of SB rounds resulted in a gradual increase in genetic gain per cycle with consistent trends among all SpeedGS schemes. Genetic gain across cycles in the first SpeedGS scenario exceeded all other scenarios for all traits except HD, as scenario one involved selection at all original breeding stages (five selection stages), while the remaining scenarios were reduced forms omitting steps. The selection for HD happened only during the SpeedGS stage, which is a common feature in all scenarios explaining its comparable gain per cycle across scenarios with the same number of SB rounds (**Figure 3**). The performance of other scenarios varied among traits depending on the number of selection stages for each trait within each scenario. Genetic gains achieved in scenario two were comparable to the first scenario for FY and Per, as both scenarios had an extra selection event for these traits after the small plots stage. Similarly, the third scenario was comparable to the first for SY and Q for which selection occurred at the single row trial stage in both scenarios. The shortest scenario (Scenario four) had the lowest genetic gain per cycle as it omitted the selection stages at small plots and single rows. Nevertheless, the performance per cycle of this scenario was equivalent or better than PS for all traits except Q.

PS outperformed all scenarios per cycle when considering GS only (SB rounds = 1) for both HD and Q (**Figure 3A**). Both traits had low selection index at the SpeedGS stage, while they were more intensely selected in the PS scheme. Having two or more SB rounds increased their genetic gains to higher levels than the phenotypic program, except for scenarios two and four for Q in which there were no single row trial. The presence of small and large effect QTL marginally affected the results of BRR-BLUP model which assumes the additive infinitesimal model. Applying GS models assuming unequal variances for QTL effects such as BayesA and BayesB did not improve genetic gain or GS accuracy for HD and Q (**Table S5**). This may be a result of the presence of large number of small effect QTL as well as increasing the frequency of the desired alleles of the large effect QTL during the first four phenotypic cycles which occurred before the first SpeedGS cycle. For example, for the majority of the replicates, between 80 to 100% of the 10 HD QTL with large effects were fixed at the commencement of the SpeedGS scenarios.

### Genetic Gain Per Year

In concordance with previous reports (e.g. Iwata and Jannink, 2011; Yabe et al., 2013; Lin et al., 2016), shortening the breeding cycle also had a large impact on boosting genetic gain per unit of time in our study. The shorter the breeding cycle, the higher the genetic gain achieved by having more SB rounds. For instance, when GS was applied with only one SB round, the genetic gains from scenario four were not significantly different from scenarios one and three for FY and Per. When increasing the SB rounds to three, the gain per year from scenario four became significantly higher than that from scenarios one and three. Conversely, the differences of genetic gains for SY and Q were significant between scenarios one and four for the scheme of one SB round (GS only), while the differences were not significant when running three SB rounds.

We also tested whether omitting the small plots and/or the single row stages resulted in any tradeoffs regarding to genetic gain. The small plots stage takes two years but it adds an extra selection step for FY and Per. Comparing scenario two with scenario four or scenarios one with scenario three, which differ only with the small plots stage, showed that there was similar genetic gain between reducing cycle time and increasing selection intensity (**Figure 4**). On the other hand, removing the single row stage, at which we select for SY and Q, resulted in significantly lower genetic gains for both traits when comparing scenarios one and three with scenarios two and four, respectively. This might be a result of the lower selection pressure proposed for SY and Q during the SpeedGS stage, which was equal to 0.15 for each trait. For this reason, if scenario four would be chosen for practical use, the selection index should be reweighted to achieve optimal gain, especially for a trait like quality that needs to be further improved in tall fescue commercial cultivars to compete with other grasses (Forster et al., 2014).

### Accuracy of Genomic Selection

Generally, the accuracies of GS achieved in our study were low for all traits. The reasons could be due to a small size of reference population (Daetwyler et al., 2008; Goddard, 2009) and inferring the genotyping of the small plots in the reference population through the mean dosage of 20 plants (Lin et al., 2016). The mean genotype of a plant population provides less resolution than single plant genotypes would. However, single plant and plot phenotypes tend to be only lowly correlated, therefore, predicting the performance of sward plants indirectly using the single plants is generally not very successful for forage yield in pasture crops including tall fescue (Waldron et al., 2008), white clover (Atwood and Garber, 1942), Kentucky bluegrass (Kramer, 1947), and alfalfa (Asay et al., 1999). On the other hand, some morphological and nutritional quality traits have moderate to high correlation between spaced and sward trials (Humphreys, 1989; Carpenter and Casler, 1990; Waldron et al., 2008). For this reason, traits that have high correlation between swards and spaced plants could have the advantage of improving their GS accuracy by phenotyping the SpeedGS crosses to be added to the reference population as those are already genotyped.

The first SpeedGS cycle had exactly the same starting reference population resulting in non-significant differences in prediction accuracy across the four scenarios. After each breeding cycle, the reference population was updated with 150 new plots (small and large) in scenario one and two or 100 large plots for scenario three and four. For this reason, the accuracy of the first two scenarios was generally higher than the other scenarios for later cycles except for HD, but, on the other hand, it requires more labor resources. The accuracy for HD prediction in scenario four was slightly higher than other scenarios mainly due to the lower number of random recombination rounds (generations) during meiosis around HD QTL, while it received no selection emphasis in any of the omitted stages. In the first three scenarios, extra selection steps happen on traits other than HD which will just randomly break the linkage disequilibrium between the genotyped SNPs and HD QTL without contributing to any sort of selective sweep around them (Kondrashov and Yampolsky, 1996). Thus, higher marker density will be beneficial to improve genomic prediction accuracy for such cases (Habier et al., 2009).

The accuracy of GS is expected to decrease when implementing more SB rounds in one cycle due to the extra crossing step shifting the population away from the reference population. Interestingly, the decrease in accuracy was not linearly correlated to the increase of the number of SB rounds. A larger decrease was observed in the scenarios with two SB rounds compared to the further decrease in the scenarios with three SB rounds for all traits across cycles. Accuracy stabilized after four SB rounds for all traits (**Figure S3**). Previous studies reported similar observations that the accuracy decays quickly during the first generations, while persisting over the following generations without updating the reference population (Habier et al., 2007; Habier et al., 2009; Sonesson and Meuwissen, 2009; Habier et al., 2010; Wolc et al., 2011). Habier et al. (2007) attributed the rapid decrease in accuracy during the first generations to the decay in genetic relationships between the reference and validation population, while the persistency of accuracy after that is due to the linkage disequilibrium between SNPs and casual mutations. Previous reports also showed that higher marker densities retained more GS accuracy after a number of breeding generations without updating the reference population (Habier et al., 2009), indicating the importance of SNP density for breeding schemes aiming to extensively depend on speed breeding.

Previous GS studies on perennial ryegrass resulted in comparable or higher prediction accuracies in comparison with our simulation. Faville et al. (2018) used a reference population of 517 individuals that have comparable quick LD decay to our population. This population is similar to our reference in breeding cycle six with SB = 1 (**Figure 5**). They found that the accuracy of HD prediction ranged between 0.4 and 0.52, which is very comparable to our results. They also estimated the accuracy of a grazing management trait (equivalent to persistency) to range from 0.07 to 0.3, which is also within our accuracy range. Their accuracy was not affected by the SNP density (40,000 vs. one million SNPs). Using a diverse reference of 364 individuals (slightly smaller than our starting reference population), Grinberg et al. (2016) achieved an average accuracy for FY of 0.15 over different phenotypic measures. Other studies that used less diverse populations achieved high accuracies. Fè et al. (2015, 2016) used a population with high relatedness and their accuracies of predicting HD, SY, and other quality traits were almost three times higher than our estimations when considering the same reference population size. Similarly, Pembleton et al. (2018) used a population with less diversity compared to our simulated population and they achieved an average accuracy of 0.76 for HD and 0.33 for FY. These values are almost double the values than in our study. Taken together, the prediction accuracies we report were in a realistic range with a possible bias towards being conservative estimates.

### Inbreeding vs. Speed Breeding

Increasing the selection rounds and intensities through SB increases the risk of running out of heterozygosity due to extensive inbreeding. This can increase the opportunity for deleterious recessive genes to become prevalent after a few cycles of breeding program and can lead to inbreeding depression (Kim et al. 2015). Previous reports showed that GS can significantly increase inbreeding (Lin et al., 2016). Our results also showed that both GS and SB can significantly increase inbreeding, and, the shorter the breeding scheme, the higher the inbreeding rate per year. However, in our simulation, we reduced the size of the initial population for the SpeedGS scheme to 1,000 (20% of that in the phenotypic scheme) to make the proposed scheme cost-effective, which could have further contributed to increasing the inbreeding rate. Additionally, we prioritized crosses using a half million potential offspring GEBVs. Increasing the number of SB rounds affected the shorter schemes more than the longer ones. For instance, the inbreeding per year for scenarios four and two (1,000 crosses × one progeny) was not significantly different for the GS only scenario, but the inbreeding for the former became significantly higher when SB rounds = 3 (**Figure 6**).

Various mathematical methods have been tested for controlling inbreeding in animal/plant breeding (Meuwissen, 1997; Pryce et al., 2012; Lin et al., 2017b; Gorjanc et al., 2018). Lin et al. (2017b) proposed a heuristic algorithm to penalize both 1) the selection of mated parents by their co-ancestry and 2) the GEBVs for the candidate offspring using their parental co-ancestry. Their proposed method resulted in only one third of the original GS scheme inbreeding rate without significant reduction in genetic gain. While such mathematical models can considerably recover the populations from inbreeding and they have been extensively investigated (Wray and Goddard, 1994; Meuwissen, 1997; Pryce et al., 2012), in this paper we tried to investigate some other breeding practices to reduce inbreeding. Our hypothesis was to test the effect of exploiting higher diversity in stages where a limited number of parents are involved. More specifically, the crosses that form the synthetic population or the initial population. Having more parents and crosses in both stages resulted in a large reduction in inbreeding rate. The improvement in the inbreeding rate did not decline when having fewer crosses during the SpeedGS stage and it did not affect genetic gain in any trait (**Figure 7**). Thus, the diversity within crosses is essential. Other strategies to reduce inbreeding involve importing cultivars from outside the breeding program (Reif et al., 2005) or introducing new variation from wild relatives (Harlan, 1976; Hajjar and Hodgkin, 2007; Jighly et al., 2018a; Jighly et al., 2019). However, introducing new non-elite materials may reduce the genetic gain on the short term but improve it on the long term.

## Conclusion

The present study has investigated the potential of utilizing both speed breeding and genomic selection (SpeedGS) in the breeding programs of allogamous crops using stochastic computer simulation. Although low prediction accuracy for different traits was reported, all proposed SpeedGS schemes outperformed the conventional phenotypic selection scheme and the higher the number of speed breeding rounds, the higher the genetic gain obtained. Persistency, which had the lowest heritability, showed the highest improvement in SpeedGS schemes over the conventional phenotypic selection program. The reference population for the first SpeedGS cycle started with 400 plots which were updated with 100/150 plots every cycle. This small number was chosen to investigate genetic gain with the minimal possible prediction accuracy when resources are limiting. The optimal utilization of SpeedGS would require plant breeders to carefully consider its impact on inbreeding. The present study showed that increasing the diversity of parents used in multiple stages of the breeding programs can significantly reduce the inbreeding gain. Other mathematical models should also be used to further reduce the inbreeding rate and ensure long-term genetic gain. Moreover, similar comprehensive studies should also be done to simulate the potential of SpeedGS in self-pollinated crops.

## Data Availability Statement

The datasets generated for this study are available on request to the corresponding author.

## Author Contributions

AJ: planned the study, ran the simulation and data analysis, developed the new QTL simulation model, and drafted the manuscript. ZL: assisted with the phenotypic selection simulation. LP, NC: provided information of tall fescue breeding program parameters. HD: planned the study, supervised the work. ZL, LP, NC, GS, BH, HD: revised the manuscript. All authors read and approved the final copy of the manuscript.

## Funding

The authors acknowledge financial support from DairyBio, a joint venture between Agriculture Victorian and Dairy Australia, and the Royal Barenbrug Group, Netherlands.

## Conflict of Interest

The authors declare that the research was conducted in the absence of any commercial or financial relationships that could be construed as a potential conflict of interest.

## Supplementary Material

Table S1 Genetic gain per cycle for all simulated breeding schemes and traits. Confidence interval (95%) indicated in brackets.

Table S2 Accuracy of genomic selection after each round of SB for all tested scenarios and traits. Standard deviations indicated in brackets. In the second row, 100 means 100 F1 × ten progenies, and 1000 means 1000 F1 × one progeny.

Table S3 Inbreeding rate increase for all simulated breeding schemes. Standard deviations indicated in brackets.

Table S4 The proportion of fixed loci after each breeding cycle for all simulated breeding schemes. Standard deviations indicated in brackets.

Table S5 Accuracy of genomic selection for HD and Q using BayesA model. Standard deviations indicated in brackets. In the second row, 100 means 100 F1 × ten progenies, and 1000 means 1000 F1 × one progeny.

Text S1 The parameter file for the software PolySim used to simulate the base population.
