# Supplementary material for: Boosting Genetic Gain in Allogamous Crops via Speed Breeding and Genomic Selection
Source: Front Plant Sci. 2019 Nov 15;10:1364. doi: 10.3389/fpls.2019.01364 (PMC6873660; doi:10.3389/fpls.2019.01364)
Supplement: Text S1 — The parameter file for the software PolySim used to simulate the base population. [file DataSheet_1.docx]

outFile [out.txt]

PrintProgress [Yes]

PrintEvery [1000]

MateSystem [1]

OffPerMate [1]

NoSnps [100000]

CommonAncestorNoGenotypes [1000]

NoGenotypes [1000 1000 1000 1000 10000]

CommonAncestorNoChr [7]

NoChr [7 7 7 14 21]

PloidyType [0]

MutationRate [0.00001]

NoGenerations [100000]

SaveGeneration [5000]

NoGeneration2PopSize [100]

SpeciationGenerations [8000 8200 15000 16000 25000]

DirectHybrid [1 2 3 1_2 12_3]

RecombinPRate [1]

Poisson [Yes]

MeanCO [1]
