# Supplementary material for: Boosting Genetic Gain in Allogamous Crops via Speed Breeding and Genomic Selection
Source: Front Plant Sci. 2019 Nov 15;10:1364. doi: 10.3389/fpls.2019.01364 (PMC6873660; doi:10.3389/fpls.2019.01364)
Supplement: Supplementary file 8 [file Image_2.pdf]

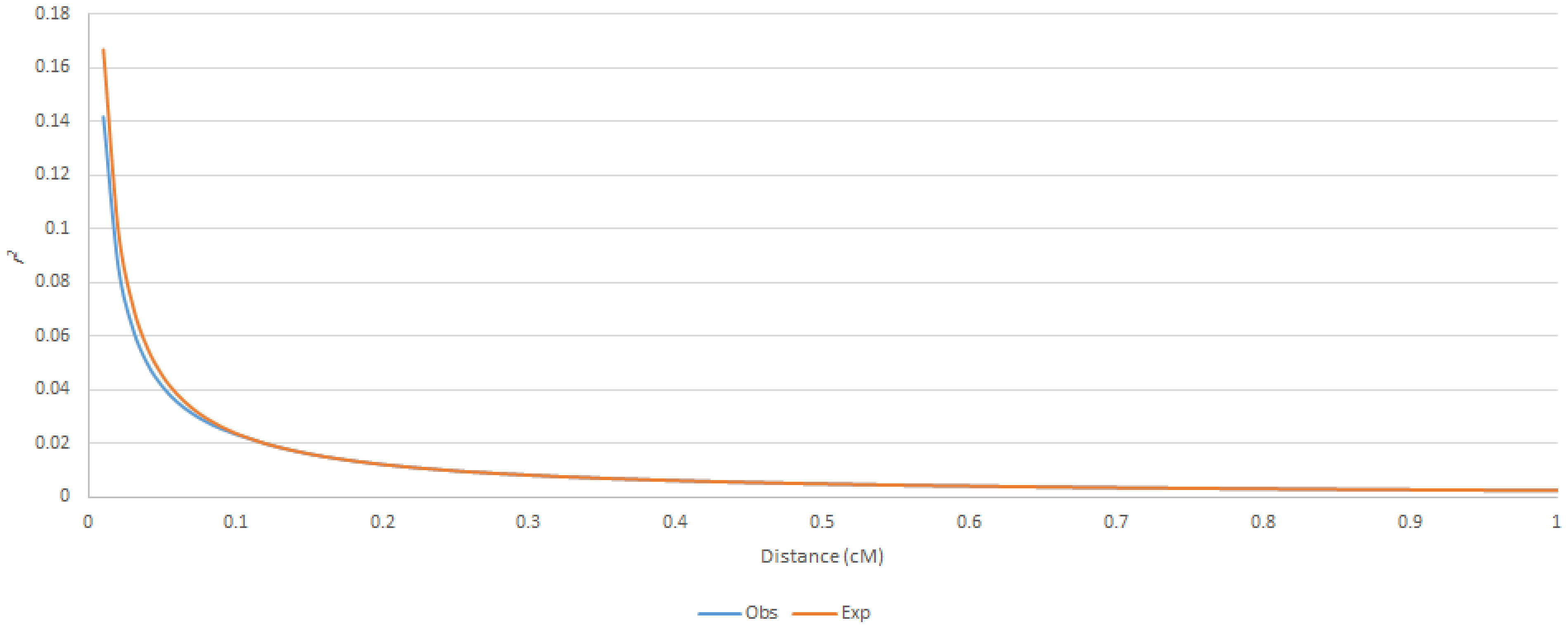

Figure S2. Comparison between the expected decay of LD and the observed one for the simulated tall fescue base population.
