# Supplementary material for: Boosting Genetic Gain in Allogamous Crops via Speed Breeding and Genomic Selection
Source: Front Plant Sci. 2019 Nov 15;10:1364. doi: 10.3389/fpls.2019.01364 (PMC6873660; doi:10.3389/fpls.2019.01364)
Supplement: Supplementary file 9 [file Image_3.pdf]

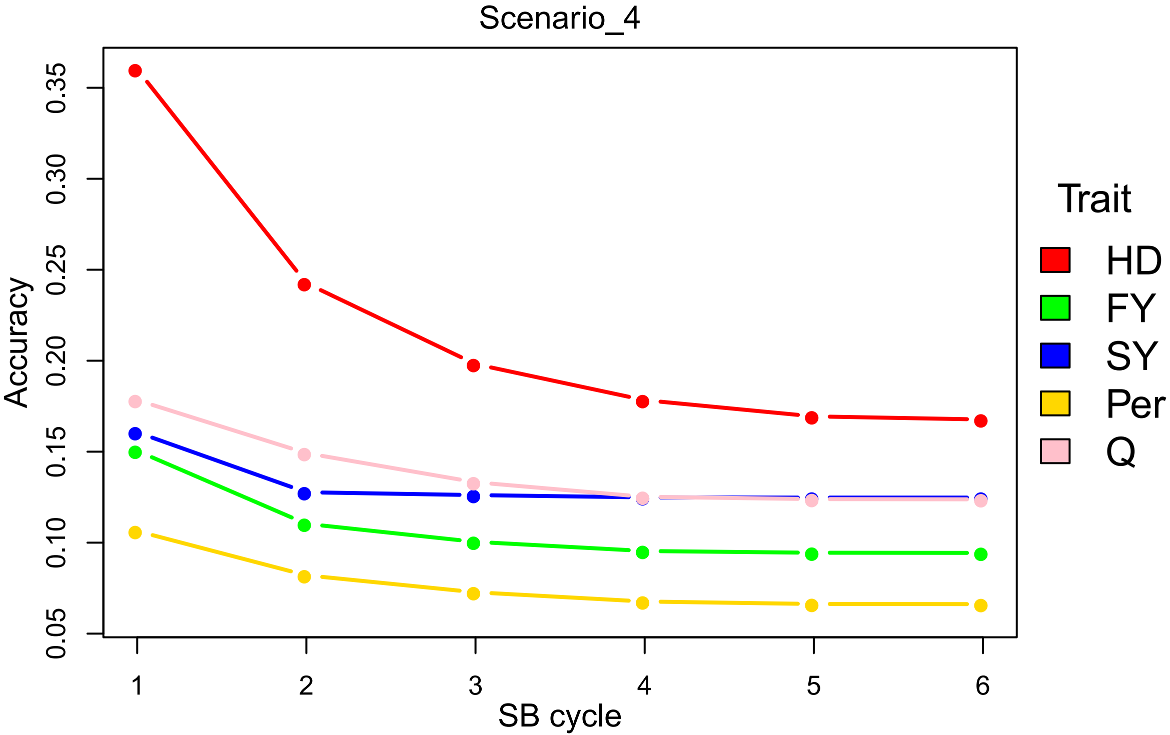

Figure S3. The changes in the accuracy of genomic selection over six SB rounds for scenario four.
